# Hebbian learning of hand-centred representations in a hierarchical neural network model of the primate visual system

**DOI:** 10.1371/journal.pone.0178304

**Published:** 2017-05-31

**Authors:** Jannis Born, Juan M. Galeazzi, Simon M. Stringer

**Affiliations:** 1 Oxford Centre for Theoretical Neuroscience and Artificial Intelligence, Department of Experimental Psychology, University of Oxford, Oxfordshire, United Kingdom; 2 Institute of Cognitive Science, University of Osnabrück, Osnabrück, Germany; Plymouth University, UNITED KINGDOM

## Abstract

A subset of neurons in the posterior parietal and premotor areas of the primate brain respond to the locations of visual targets in a hand-centred frame of reference. Such hand-centred visual representations are thought to play an important role in visually-guided reaching to target locations in space. In this paper we show how a biologically plausible, Hebbian learning mechanism may account for the development of localized hand-centred representations in a hierarchical neural network model of the primate visual system, VisNet. The hand-centered neurons developed in the model use an invariance learning mechanism known as *continuous transformation* (CT) learning. In contrast to previous theoretical proposals for the development of hand-centered visual representations, CT learning does not need a memory trace of recent neuronal activity to be incorporated in the synaptic learning rule. Instead, CT learning relies solely on a Hebbian learning rule, which is able to exploit the spatial overlap that naturally occurs between successive images of a hand-object configuration as it is shifted across different retinal locations due to saccades. Our simulations show how individual neurons in the network model can learn to respond selectively to target objects in particular locations with respect to the hand, irrespective of where the hand-object configuration occurs on the retina. The response properties of these hand-centred neurons further generalise to localised receptive fields in the hand-centred space when tested on novel hand-object configurations that have not been explored during training. Indeed, even when the network is trained with target objects presented across a near continuum of locations around the hand during training, the model continues to develop hand-centred neurons with localised receptive fields in hand-centred space. With the help of principal component analysis, we provide the first theoretical framework that explains the behavior of Hebbian learning in VisNet.

## 1 Introduction

Reaching for objects and manipulating them with our hands is one of the most natural abilities of primates and a fundamental way of interacting with our environment. We can learn to coordinate our limbs and hands elegantly, dexterously and almost effortlessly, yet implementing this level of skillfulness in artificial systems or robotic platforms still poses a challenging problem [1]. Part of the problem of reaching for an object in space involves how we represent the location of such a target object relative to the body or the relevant part of the body. Neuroscientific studies seem to suggest that the brain represents the location of objects using a rich tapestry of coordinate frames [2–7]. Investigating the reference frames of neurons performing, guiding or preparing motor actions is among the most prominent issues of current neuroscience. It is crucial for understanding how spatial information is processed and represented in the brain in order to facilitate the planning of movements within our peripersonal space.

While visual information is initially represented in retino-centric coordinates, precise guiding of motor tasks such as visually-guided reaching is enabled by non-retinal coordinate maps connected to various body parts in the later stages of processing. The occipito-parietal components of the dorsal stream connect via the parieto-premotor pathway to various regions of the posterior parietal cortex (PPC) particularly to areas within the intraparietal sulcus (IPS) and the superior parietal lobe [8]. In general, the parieto-premotor pathway seems to perform the necessary coordinate transforms required for planning and guiding motor tasks [9]. Neurons encoding the location of objects in a head-centred, limb-centred, body-centred, hand-centred frame of reference or mixtures of the latter two have been reported [10, 11]. Single cell recordings in non human primates generally reveal a gradient in response properties from predominantly eye-centred encoding in early visuomotor areas to body-centred or effector-centred representations in more parieto-frontal areas [12, 13], including hand-centred representations of reach vectors found in area 5d [3, 14]. Neurons with hand-centred receptive fields fire maximally whenever a target object is at a specific position with respect to the hand while remaining relatively invariant against head movements, saccades or even across different positions of the hand relative to the body. Hand-centred encoding is not restricted to area 5d but has been found across different parts of the PPC and premotor cortex [15–17]. While in ventral premotor cortex (PMv) hand-centred representations independent from eye position have been reported [18, 19], in dorsal premotor cortex (PMd) neurons encoding reference frames centered on the hand, eye or a combination of both have been found [20, 21]. Recently, studies have also identified and studied the role of hand-centered representations in the human brain [22–26].

The aim of this paper is to present a new model of how visual neurons with hand-centred receptive fields may develop in the primate brain using biologically plausible learning mechanisms. Various computational models have been previously suggested to account for the different stages of coordinate transformations, aiming to explain some of the response properties found in neurophysiological recordings. For example, an early model developed by Zipser and Andersen [27] showed how eye-centred visual representations could be mapped to head-centred representations by supervised learning in a backpropagation of error network. Later, Chang et al. [28] adapted the model of Zipser and Andersen by adding a proprioceptive hand-position signal, which allowed the development of hand-centred responses in the output layer. However, these two models made use of supervised backpropagation of error learning, which is generally regarded as biologically implausible for a couple of reasons [29]. For example, it is difficult to explain where a suitable teaching signal might come from to guide supervised learning of coordinate transformations from one reference frame (e.g. eye-centred) to another (e.g. hand-centred) in the visual system. Moreover, the propagation of the teaching signal down to earlier layers is regarded as biologically implausible because the feedback neurons would require precise knowledge of the derivatives (with respect to the target signal) of the feedforward neurons exactly at the operating time. For these reasons, we propose that a more biologically plausible approach for explaining the development of coordinate transformations in the primate visual system is to build neural network models that self-organise their synaptic connectivity through an unsupervised process of visually-guided competitive learning. However, progress with such model architectures has been fairly scarce. In an extensive approach to accounting for the neural mechanism underlying coordinate transforms, Pouget and Sejnowski [30] modelled the response properties of neurons in PPC by basis function units that could represent multiple reference frames simultaneously. Recently, Magosso et al. [31, 32] proposed an unsupervised neural network model that integrates visual and tactile peri-hand representations to generate bimodal hand-centred neurons. Nevertheless, these authors did not provide a theory of how hand-centred visual neurons might arise in the primate brain because they assumed their existence in early layers of the network.

Another study was recently carried out by Galeazzi et al. [33], who modelled the visually-guided development of hand-centred visual neurons using an unsupervised, competitive learning mechanism. These authors demonstrated the development of neurons that responded selectively to visual objects presented in particular locations with respect to the hand, while responding invariantly as the hand-object configuration was shifted across different retinal locations. It was subsequently shown that this unsupervised learning model was sufficiently robust that it could still develop hand-centred visual representations even when being trained with more realistic images of the hand presented against natural scenes [34] or when being trained on image sequences that were driven by natural human gaze changes recorded with an eyetracking system [35]. These model simulations all relied on an invariance learning mechanism known as *trace learning* [36]. This learning mechanism operates by binding together input patterns that tend to occur in temporal proximity. If the eyes saccade around a visual scene containing a fixed hand-object configuration during visual training, then trace learning causes the corresponding images of that hand-object configuration in different retinal locations to become associated with the same subset of output neurons. These neurons are then able to respond selectively to the particular hand-object configuration in a shift invariant manner across the different retinal locations seen during training.

However, the trace learning mechanism used by Galeazzi et al. [33] has a couple of potential limitations from the point of view of biological plausibility. First, the trace learning rule used to modify the synaptic weights needs to incorporate a memory trace of recent neuronal activity, and so it is significantly more complex than a standard Hebbian learning rule. Secondly, even more limiting is the fact that trace learning requires that different retinal views of the same hand-object configuration must be seen clustered close together in time. That is, each hand-object configuration must be thoroughly visually explored through a sequence of saccadic shifts in retinal position before encountering the next hand-object configuration. If these image sequence statistics are compromised during training, for example if different hand-object configurations are seen in quick succession, then this will sharply degrade the ability of the model to develop hand centred visual neurons. This may occur if the relative positions of the hand and nearby objects are changing rapidly, which will happen during reaching for example. This weakness of trace learning was demonstrated in the original study of Stringer et al. [37], which showed trace learning failing when two different visual stimuli were rapidly interleaved through time. These limitations of the trace learning mechanism employed by Galeazzi et al. [33] beg the question of whether an alternative learning mechanism might be found that can produce hand-centred visual neurons using a simpler Hebbian learning rule and without the restrictive assumption that particular hand-object configurations must be visually explored through a lengthy series of saccades before a new hand-object configuration comes into view.

In this paper, we present a new unsupervised, competitive learning model of how hand-centred visual neurons may develop, which eliminates the two potential weaknesses of trace learning described above. This simplified approach utilises an invariance learning mechanism known as *continuous transformation (CT) learning*, which was successfully applied to the problem of rotation invariant visual object recognition by Stringer et al. [37] and to guide translation invariant visual representations by Perry et al. [38]. CT learning relies on a standard Hebbian learning rule, with no need for a memory trace of recent neuronal activity. The learning mechanism operates by binding together subsets of smoothly changing input patterns, for example, corresponding to particular hand-object configurations seen shifted across different retinal locations due to saccades. This binding is achieved by exploiting the spatial overlap that naturally occurs between similar images such as the same hand-object configuration presented in nearby retinal locations. Details of the CT learning mechanism and its performance properties are described in [37]. Importantly, CT learning does not require that the input patterns associated together (e.g. different retinal views of the same hand-object configuration) are seen in close temporal succession. In other words, CT learning is not limited by constraints on the temporal sequencing of training images. Our simulations described below confirm that this learning mechanism is robust with respect to the order of stimulus presentation. CT learning may consequently cope with a much broader range of natural spatiotemporal image statistics, in which the relative positions of the hand and surrounding objects do not remain fixed for relatively long periods. Regarding generalization, we can further report that our model responds properly to untrained hand-object configurations given that the density of initial training across the hand-centred space was sufficiently high.

Furthermore, to verify the robustness of this learning mechanism to more realistic training conditions, we increased the density of trained hand-centred locations from almost orthogonal locations in the first simulations (e.g. *left*, *top*, *right* around the hand) to a near continuum (i.e. >10 locations). This poses a potential challenge for CT learning given its inherent learning properties. CT learning operates by simply binding together subsets of smoothly varying input patterns by exploiting the spatial overlap or similarity between successive members. However, by increasing the density of trained hand-centred locations, neighbouring locations start to show a greater degree of overlap, making it more difficult to separate different views of highly overlapping hand-object configuration. The question is then, how would CT learning behave in this scenario. First, CT learning could continue to bind together different retinal views of the same (or neighbouring) hand-object configurations to produce neurons with hand-centred receptive fields. Alternatively, CT learning could also start binding together overlapping input patterns corresponding to different hand-object configurations at the same retinal location, and therefore fail to produce hand-centred representations that respond selectively to a highly localised region of hand-centred space. Indeed, our simulations showed that the network continued to develop hand-centred neurons even as the number of trained hand-centred locations approached a continuum. In this case, individual hand-centred neurons learn to respond to a small localised region of hand-centred space across different retinal locations. Importantly, we find that the receptive fields of these hand-centred neurons are distributed uniformly through the hand-centred space.

In the final results section of this paper, we provide a theoretical framework for explaining why CT learning continues to drive the development of hand-centred neurons even when the network is trained on a (near) spatial continuum of hand-centred target locations. This is achieved by applying Principle Component Analysis (PCA) to the visual stimulus sets, consisting of alternative hand-object configurations presented across different retinal locations, which are used to train the network. It is found that the main directions of variance within the training stimuli can predict whether or not the network develops hand-centred neurons using CT learning. Most importantly, this new framework carries implications of various previous work that utilised the same learning mechanism for translation or rotation invariant object recognition as well as differentiating face identity from face expression [37–39].

## 2 Methods

### 2.1 VisNet

**Fig 1:**
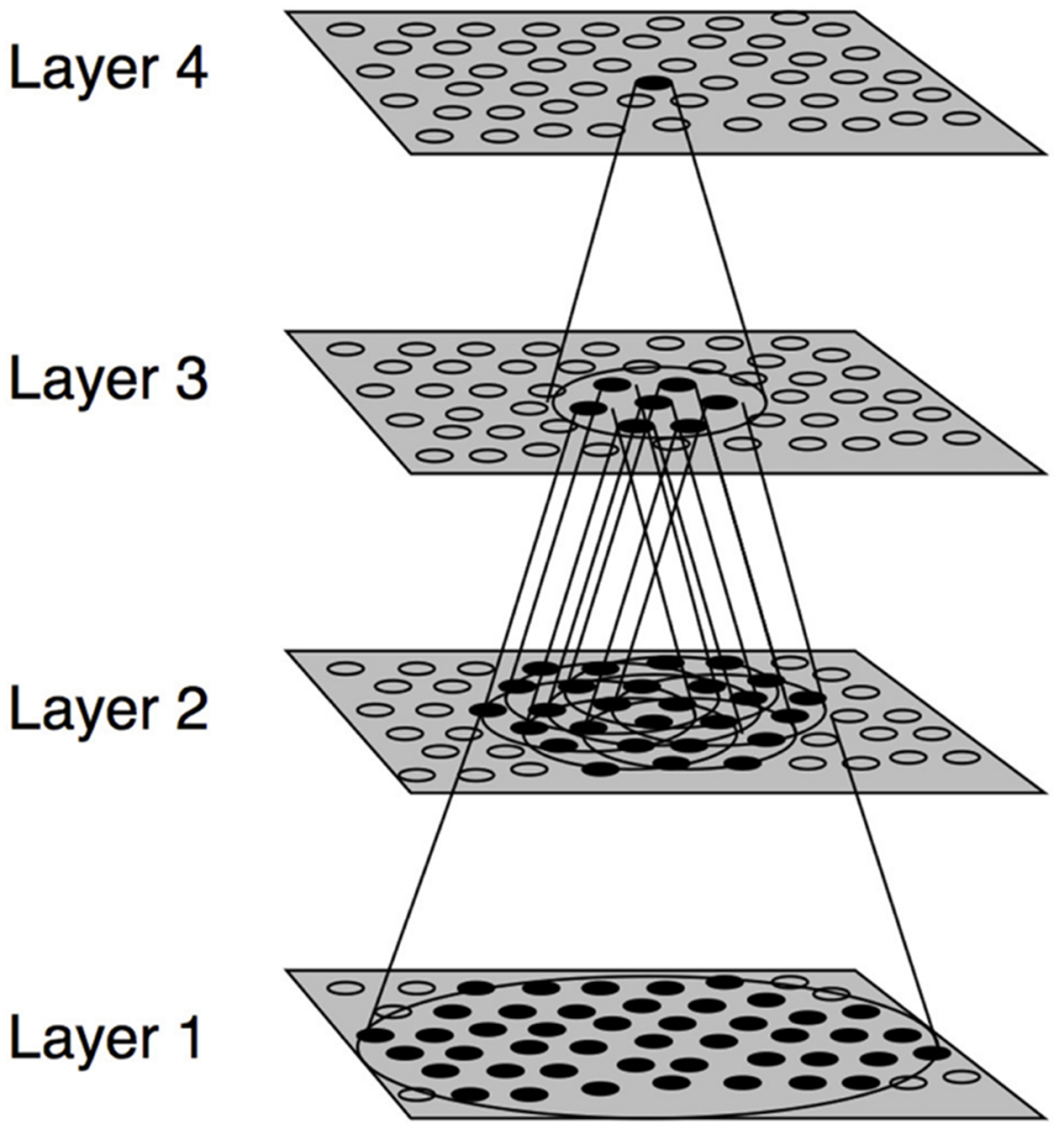
VisNet architecture. Stylized image of the four-layered VisNet neural network architecture. The architecture of the network shows a competitive, hierarchical organization broadly reflecting that observed in the primate dorsal visual system. Convergence through the network is designed to provide fourth-layer neurons with information from across the entire input retina. Layer 4 corresponds to the output layer, in which hand-centred visual neurons develop.

The numerical simulations presented in this paper were conducted using the VisNet model of hierarchical processing in the primate visual system, which is shown in Fig 1. The same VisNet architecture was used in our previous simulations of the development of hand-centred visual neurons using trace learning [33–35]. However, in the simulations reported below, we implement a Hebbian learning rule in the model instead of the trace rule.

VisNet is composed of a hierarchy of four competitive layers of neurons. Within each layer, the neurons compete with each other through lateral inhibitory mechanisms described below. Layer 4 is the output layer, which develops hand-centred visual neurons after training. Between successive layers of neurons there are bottom-up (feed-forward) synaptic connections, which are modified by a Hebbian learning rule during training. This hierarchical network architecture loosely represents the hierarchical organisation of the primate dorsal visual pathway.

The forward connections between the retinal layer and the first layer of the network are derived from a topologically corresponding region of the preceding layer, using a Gaussian distribution of connection probabilities. These distributions are generated by a radius containing approximately 67% of the connections from the preceding layer. The connection probabilities for later layers are derived from a uniform distribution across all neurons from the preceding layer. This leads to an increase in the receptive field (RF) size of neurons through successive layers of the network hierarchy. The network dimensions used for this study are shown in Table 1.

**Table 1:** Network dimensions. Table shows number of neurons per layer, number of synapses to preceding layer and size of receptive field from which the connections are received.

	Dimensions	# of Connections	RF radius
Layer 4	32 × 32	100	16
Layer 3	32 × 32	100	16
Layer 2	32 × 32	100	16
Layer 1	32 × 32	100	9
Retina	128 × 128 × 16	-	-

The natural visual images of size 128 × 128 pixels are pre-processed by an initial layer mimicking the response properties of V1 simple cells, wherein each (*x*, *y*)-location contains a bank of Gabor filter outputs corresponding to a hypercolumn generated by

$$\begin{array}{c} g\left(x,y;\lambda ,\theta ,\psi ,\sigma ,\gamma \right)=\exp\left(-\frac{x^{\prime 2}+\gamma^{2}y^{\prime 2}}{2\sigma^{2}}\right)\cos\left(2\pi \frac{x^{\prime }}{\lambda }+\psi \right) \end{array}$$ (1)

$$\begin{array}{ccc} x^{\prime } & = & x\cos\theta +y\sin\theta \end{array}$$ (2)

$$\begin{array}{ccc} y^{\prime } & = & -x\sin\theta +y\cos\theta \end{array}$$ (3)

for all combinations of *λ* = 16, *γ* = 0.5, *σ* = 0.56*λ*, *θ* ∈ {0, *π*/4, *π*/2, 3*π*/4} and *ψ* ∈ {0, *π*, −*π*/2, *π*/2}.

Subsequently the filtered images are fed into the first layer of the network architecture. The activation *h*_*i*_ of each neuron *i* in the network is defined as a linear sum of the inputs *y*_*j*_ from afferent neurons *j* in the previous layer weighted by the corresponding synaptic weights *w*_*ij*_. That is,

$$\begin{array}{c} h_{i}=\sum_{j}w_{ij}y_{j} \end{array}$$ (4)

where *y*_*j*_ denotes the firing rate of the presynaptic neuron *j*, and *w*_*ij*_ the synaptic strength from neuron *j* to neuron *i*.

Within each layer competition is implemented in the following two stages and is graded rather than winner-take-all.

First, to implement lateral inhibition the activations of neurons within a layer are convolved with a spatial filter *I*, where *δ* controls the contrast, *σ* controls the width, and *a* and *b* index the distance to the centre of the filter.

$$\begin{array}{c} I_{a,b}=\{\begin{array}{cc} -\delta e^{-\frac{a^{2}+b^{2}}{\sigma^{2}}} & \text{if}\;a\neq 0\;\text{or}\;b\neq 0,\\ 1-\sum_{\begin{array}{c} a\neq 0\\ b\neq 0 \end{array}}I_{a,b} & \text{if}\;a=0\;\text{and}\;b=0. \end{array} \end{array}$$ (5)

Typical lateral inhibition parameters are given in Table 2.

**Table 2:** Lateral inhibition parameters.

Layer	1	2	3	4
Radius, *σ*	1.38	2.7	4.0	6.0
Contrast, *δ*	1.5	1.5	1.6	1.4

Next, contrast enhancement is applied by means of a sigmoid activation function

$$\begin{array}{c} y=f^{sigmoid}\left(r\right)=\frac{1}{1+e^{-2\beta (r-\alpha )}} \end{array}$$ (6)

where *r* is the activation after lateral inhibition, *y* the firing rate after contrast enhancement, and *α* and *β* the sigmoid’s threshold and slope respectively. The parameters *α* and *β* are constant within each layer, although *α* is adjusted to control the sparseness of the firing rates. For the simplified case of neurons with binarised firing rates ∈ {0, 1}, the sparseness of the firing within a layer is the proportion of neurons that are active. So, for example, in order to achieve a sparseness of 5%, the threshold *α* is set to the value of the 95th percentile point of the activations within the layer. Typical parameters for the sigmoid activation function are shown in Table 3.

**Table 3:** Sigmoid parameters. The sigmoid parameters used to control the global inhibition within each layer of the model.

Layer	1	2	3	4
Percentile	99.2	98	88	90
Slope *β*	190	40	75	26

At the beginning of training, the synaptic weights throughout the network are initialised to random values. These synaptic weights are then updated each time an image of particular hand-object configuration in a specific retinal position is presented to the network. It is these learning updates to the synaptic weights that drive the development of hand-centred neuronal responses in the output layer. In previously published work, we used a trace learning rule to model the development of hand-centred receptive fields [33–35]. However, in the new simulations with CT learning reported below, the synaptic connections between successive layers of neurons are updated using the Hebbian learning rule

$$\begin{array}{c} \Delta w_{ij}=\alpha y_{i}y_{j} \end{array}$$ (7)

where *y*_*j*_ is the firing rate of presynaptic neuron *j*, *y*_*i*_ is the firing rate of postsynaptic neuron *i*, and *α* is the learning rate which is set to 0.1. In order to limit the growth of each neuron’s weight vector ***w***_***i***_, synaptic scaling is applied. To regulate the total synaptic input driving each neuron, while maintaining the relative strengths of individual synapses on the neuron’s dendrites, the length of each neuron’s synaptic weight vector is renormalized at the end of each timestep during training by setting

$$\begin{array}{c} \sqrt{\sum_{j}w_{ij}^{2}}=1 \end{array}$$ (8)

as usual in competitive learning [40]. This computational step is in accordance with neuronal recordings by Turrigano et al. [41] that showed synaptic learning to be accompanied by synaptic scaling as homeostatic regulation. In a computational model, Lazar et al. [42] showed absence of weight vector renormalization caused neurons to encode redundant information by synchronizing their firing patterns.

#### 2.1.1 Relation to HMAX Model

VisNet has been often compared to HMAX, another computational model of the visual cortex that was established by Riesenhuber and Poggio [43] and further improved by Serre [44]. Like VisNet, HMAX aims for biological plausibility and utilises an unsupervised, hierarchical structure to find a suitable trade-off between selectivity and invariance in visual recognition. Typically, HMAX contains by a factor 100 more computational units than VisNet (65,536 for a 128 × 128 retina on all 4 layers). In HMAX, the final layer’s output is usually fed into a non-biologically plausible support vector machine or least squares algorithm for task classification, whereas VisNet uses Shannon information theory measures, based on dot product encoding, that are likely to occur in the brain in a similar fashion [45]. While VisNet is usually trained with image sequences as they transform in the world (such that rotation, view, translation and illumination invariance can be learned), HMAX is dominantly trained with images from large databases that seek for object classification. In a detailed comparison, Rolls and Robinson [46, 47], revealed that object classification works rather poor with both approaches (compared to backpropagation approaches) and that VisNet develops invariant representations that generalise to unseen transforms (not so for HMAX). Most importantly, VisNet did not respond to scrambled faces when trained on proper faces, suggesting the network to encode shape information, whereas HMAX retained stable firing rates for scrambled and unscrambled faces, suggesting the network to encode low-level features such as texture or particular facial spots.

### 2.2 Continuous transformation learning hypothesis

**Fig 2:**
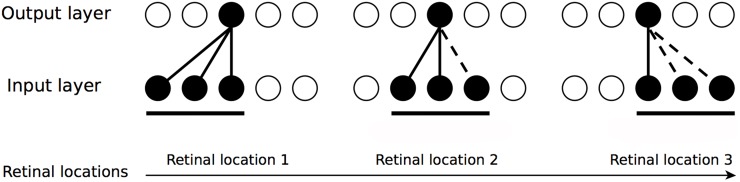
CT learning hypothesis. Basic principle of CT learning illustrated in a simplified network with a single layer of bottom-up synaptic connections between two layers. This illustration provides an idealized example how presenting the same hand-object configuration across spatially overlapping retinal locations drives neurons in the output layer to respond to this configuration for two similar retinal locations (leftmost and middle illustration). This process can be continued for subsequent shifts, provided that a sufficient proportion of input cells stay active between individual shifts (rightmost illustration). Figure adapted from Stringer et al. [37].

Established by Stringer et al. [37], CT learning is an unsupervised competitive learning mechanism relying on spatial similarity of successive transforms of a visual stimulus, for instance, as it smoothly shifts across the retina during a sequence of saccades. An idealized example of how CT learning guides the development of neurons with localized, hand-centred receptive fields in VisNet shows Fig 2.

The operation of CT learning is shown in a simplified network architecture with a single layer of bottom-up synaptic connections between an input layer and an output layer. The neurons in the input layer may be taken to provide an idealised visual representation of a particular spatial configuration of the hand and a nearby object. When such an input pattern is presented to the network, activation is evoked in a subset of neurons in the input layer. Then activation is transmitted through the initially random forward connections to the output layer, where one (or more) of the output neurons wins the competition and fires (shaded neuron). Next, the synaptic weights between active neurons in the input and output layers get strengthened according to the Hebbian learning rule (shown as solid lines between the layers). A gradual shift in the position of the visual stimulus (i.e. a hand-object configuration) across the retina will activate a sequence of spatially overlapping input patterns. Next, the activation of some input neurons with previously strengthened synaptic weights will drive the activation of the same output neuron and make it more likely to win the competition again. Consequently, the synaptic weight between this output neuron and the newly active input neuron(s) will get strengthened (dashed line). This procedure is repeated again and again as the visual stimulus shifts across the retina due to saccades during training. As a result, after training, the same output neuron will have learned to respond to the particular hand-object configuration across different retinal locations due to the high degree of spatial overlap between these images.

In contrast to trace learning, it is important to emphasise that CT learning does not require that each spatial configuration of the hand and a target object is seen with temporal continuity as it shifts across the retina. In other words, CT learning can still bind together different retinal views of the same hand-object configuration even if different hand-object configurations are seen rapidly interleaved with each other through time. CT learning thus potentially offers a far more robust mechanism than trace learning for the development of hand-centred neurons, in that it can cope with a broader, more natural range of visual training sequences, in which the relative positions of the hand and nearby objects are sometimes changing rapidly as well as remaining stationary for a long period of time.

Furthermore, although the explanation of CT learning given above and illustrated in Fig 2 would seem to imply that each hand-object configuration needs to be shifted continuously in small steps across the retina, it was shown by Stringer et al. [37] that the learning mechanism is much more flexible than this. In fact, stimulus representations that are invariant to shifts in retinal location can still develop even if the same hand-object configuration is not shown in any two consecutively presented frames, like for example a moving object passing a static hand. In this case, output neurons may continue to develop (retinal) location invariant responses as long as the hand-object configuration is presented to the network across the entire space of overlapping retinal positions over the total training session.

A related mechanism to CT learning, Slow Feature Analysis (SFA), has been shown to extract slowly varying features from a sequence of images to form translation or rotation invariant representations [48]. As we suggest in this paper, CT learning drives the network to encode information along the directions of highest variance (i.e. to approximate a PCA), whereas SFA finds principal components with minimal eigenvalues (i.e. least variance). Importantly, SFA utilises the fact that the sensory signal of any photoreceptor varies quickly while the environment varies comparably slowly; thus it is the temporal structure of the signal determining the result of the algorithm. In contrast to that, CT learning allows for interleaving since it exploits purely the spatial overlap of the input signal, irrespective of any temporal component as shown in section 3.3. Therefore, as long as a feature has higher correlation within different views of the same hand-object configuration than in views of different hand-object configurations, then it is more likely to be represented by the same neuron. This allows to develop invariant representations not only if the related feature slowly varies over time but entirely independent of the temporal dimension. Accordingly, SFA seems to be more closely related to trace learning than to CT learning. However, unlike the latter two learning mechanisms, that are based on Hebbian learning, SFA is not regarded as biologically plausible because there is no evidence suggesting SFA to be computed by neurons in the brain.

### 2.3 Network performance analysis

We developed techniques for analysing the response characteristics of neurons using two different methodologies. In the first approach, we tested whether neurons in the output layer had learned to encode a finite number of discrete hand-centred locations, with individual neurons responding to only one of these hand-centred locations regardless of the retinal position of the hand-object configuration.

However, a population of hand-centred neurons in the brain needs to cover a continuous space of such hand-centred locations, with individual neurons having a finite sized receptive field covering some small region of the hand-centred space. Therefore, our second approach treated the hand-centred space as a spatial continuum, with the receptive fields of individual hand-centred neurons covering a small region of this space. We describe the mathematical details of these two analytical approaches next.

#### 2.3.1 Analysis based on discrete hand-centred locations

In our first approach to analysing the response characteristics of output neurons we test how well they have learned to represent a finite number *N* (up to ten) of discrete hand-centred locations across different retinal locations. Each ideal hand-centred neuron should respond to only one of the hand-centred locations, and should respond to that hand-centred location across all retinal positions of the hand-object configuration. In order to assess how well the output neurons represent a finite number *N* of discrete hand-centred locations, we utilize two information theory measures, single and multiple cell information. Both have been applied previously to analyse the performance of the VisNet model, and were introduced by Rolls and Millward [49]. In this study, we define a stimulus as a particular hand-object configuration.

Whenever an output neuron responds to a particular hand-object configuration across all retinal locations, but does not respond to any other hand-object configuration, then this neuron will convey maximal single cell information. The amount of single cell information carried by a specific neuron about a particular hand-object configuration is computed by

$$\begin{array}{c} I\left(s,R\right)=\sum_{r\in R}P\left(r|s\right)\log_{2}\frac{P(r|s)}{P(r)} \end{array}$$ (9)

where the stimulus-specific information *I*(*s*, *R*) is the amount of information the set of responses *R* of a single cell has about a specific stimulus *s* (i.e. target location with respect to the hand), while the set of responses *R* corresponds to the firing rate *y* of a cell to each of the hand-object configurations presented in all retinal locations. The maximum single cell information possible is

$$\begin{array}{c} \text{Max.}\;\text{single}\;\text{cell}\;\text{information}={\text{log}}_{2}\left(N\right), \end{array}$$ (10)

where *N* is the number of different spatial configurations of the hand and target object. For example, with *N* = 8 hand-object configurations, the maximum single cell information is 3 bits.

In case a neuron does not respond to its preferred hand-object configuration across all retinal locations or in case it responds to another hand-object configuration, then the amount of single cell information carried by that neuron will not reach the theoretical limit. Thus, single cell information is a useful measure for assessing how hand-centred the response characteristics of a neuron are, with maximal single cell information implying that the neuron displays perfect hand-centred responses.

Nevertheless, it does not address the question whether all *N* hand-object configurations are represented by the population of output neurons. For this reason, the multiple cell information computes the average amount of information about which hand-object configuration was presented on the basis of the responses of the most informative output cells. This procedure is used to verify whether, across the entire population of output cells, there is information about all of the different hand-object configurations that have been shown. Methods for calculating the multiple cell information measure have been previously described by Rolls and Millward [49]. In brief, from a single presentation of a stimulus (i.e. hand-object configuration), we calculate the average amount of information obtained from the responses of all the cells regarding which stimulus is shown. This is achieved through a decoding procedure that estimates which stimulus *s*′ gives rise to the particular firing rate response vector on each trial. A probability table of the real stimuli s and the decoded stimuli *s*′ is then constructed. From this probability table, the mutual information is calculated as

$$\begin{array}{c} I\left(S,S^{\prime }\right)=\sum_{s,s^{\prime }}P\left(s,s^{\prime }\right)\log_{2}\frac{P(s,s^{\prime })}{P\left(s\right)P\left(s^{\prime }\right)}. \end{array}$$ (11)

Multiple cell information values are calculated for the subset of output cells which, according to the single cell information analysis, have the most information about which stimulus (hand-object configuration) is shown. In particular, the multiple cell information is calculated from 5 cells for each stimulus that had the most single cell information about that stimulus. Previous research has found this to be a sufficiently large subset of cells to inform the multiple cell information measure [50].

All results for single and multiple cell information measures presented in the paper are averages across 10 simulations with different random seeds used to initialise the network synaptic connectivity and synaptic weights.

#### 2.3.2 Analysis based on a continuum of hand-centred locations

The single and multiple cell information described above suffer from the deficiency that they assume a finite number of discrete hand-centred locations as well as equidistant differences between all pairs of these stimuli. While a cell is computed as carrying maximal information if it responds selectively to one hand-centred location, responses for neighboring hand-centred locations are not taken into consideration. However, of course, neurons in the brain must represent a spatial continuum of target locations in hand-centred space. In this case, individual neurons respond to a small localised region of hand-centred space rather than a single point. Therefore, in our second approach to analysing the response characteristics of output neurons we verify its generalisation abilities by increasing the number of tested hand-object up to 30 highly overlapping hand-centred locations. Therefore, we introduce a method to quantify how well each cell has learned to represent a localised region of hand-centred space across different retinal locations.

We classify a neuron in the output layer to be hand-centred using the following steps. First, we compute the firing rate responses of the neuron to each tested hand-object configuration presented in every retinal position. This gives a response matrix for the neuron, with one dimension being the hand-centred location of the target object and the other dimension being the retinal position of the whole hand-object configuration. Next, the response matrix for the cell is binarised by thresholding the firing rate responses. That is, if the firing rate response of the neuron is below the threshold *T* = 0.5 then it’s response is set to zero, otherwise it is set to one. Based on the resulting binary response matrix, also shown further below, a neuron is classified as hand-centred if it fulfils the following two criteria:

1. The neuron displays a single localised region of response within its binary response matrix.
2. The neuron displays an elongated response profile, whereby it responds to a relatively small region of the hand-centred space of target locations while responding to a much larger region of the retinal space for those preferred hand-object configurations. This is assessed using the following inequality:
  $$\begin{array}{c} \sqrt{\frac{1}{n-1}\sum_{i=1}^{n}{\left(x_{i}-\bar{x}\right)}^{2}}⩾\lambda \cdot \sqrt{\frac{1}{n-1}\sum_{i=1}^{n}{\left(y_{i}-\bar{y}\right)}^{2}} \end{array}$$ (12)
  where *n* is the total number of combinations of hand-centred target location *x*_*i*_ and retinotopic location of the whole hand-object configuration *y*_*i*_ that activate the cell, and *λ* is an adjustable parameter controlling the sensitivity of the criterion. Both, *x* and *y* dimensions are normalized in the range of [0, 1]. Examples of neurons with such elongated response fields are depicted further below in section 3.4. For all simulations *λ* was set to 4.

Although these criteria can identify a population of hand-centred output neurons, this does not inform us about whether all local subregions of the hand-centred space of target locations are represented equally. Does the output population learn to represent the entire space of hand-centred target locations evenly, or does the network completely fail to represent some local subregions? In order to investigate this question, we carried out a further stage of analysis, which counts the amount of hand-centred neurons per training location, as follows.

Each identified hand-centred neuron contributes to each location by the relative invariance of its response to that configuration over all retinal locations. For example, if a hand-centred neuron responded to all 10 retinal locations of one particular hand-object configuration and 5 retinal locations of a neighbouring configuration, then it is considered to be 67% and 33% hand-centred for each of those configurations, respectively. These values are computed for all identified hand-centred output neurons, and then used to assess the degree of coverage of the hand-centred space of target locations by the entire output population. Ideally, every location along the hand-centred space should be represented by approximately the same amount of hand-centred neurons.

### 2.4 Training procedure

During training, VisNet was presented with greyscale images of 128 × 128 pixels consisting of a hand and a single, black circular target object (diameter = 36 pixels) at a specific position relative to the hand. Throughout all simulations, the position of the hand moves across the retina while the orientation of the hand is kept constant (i.e. simulations focus on learning translation rather than rotation invariance).

**Fig 3:**
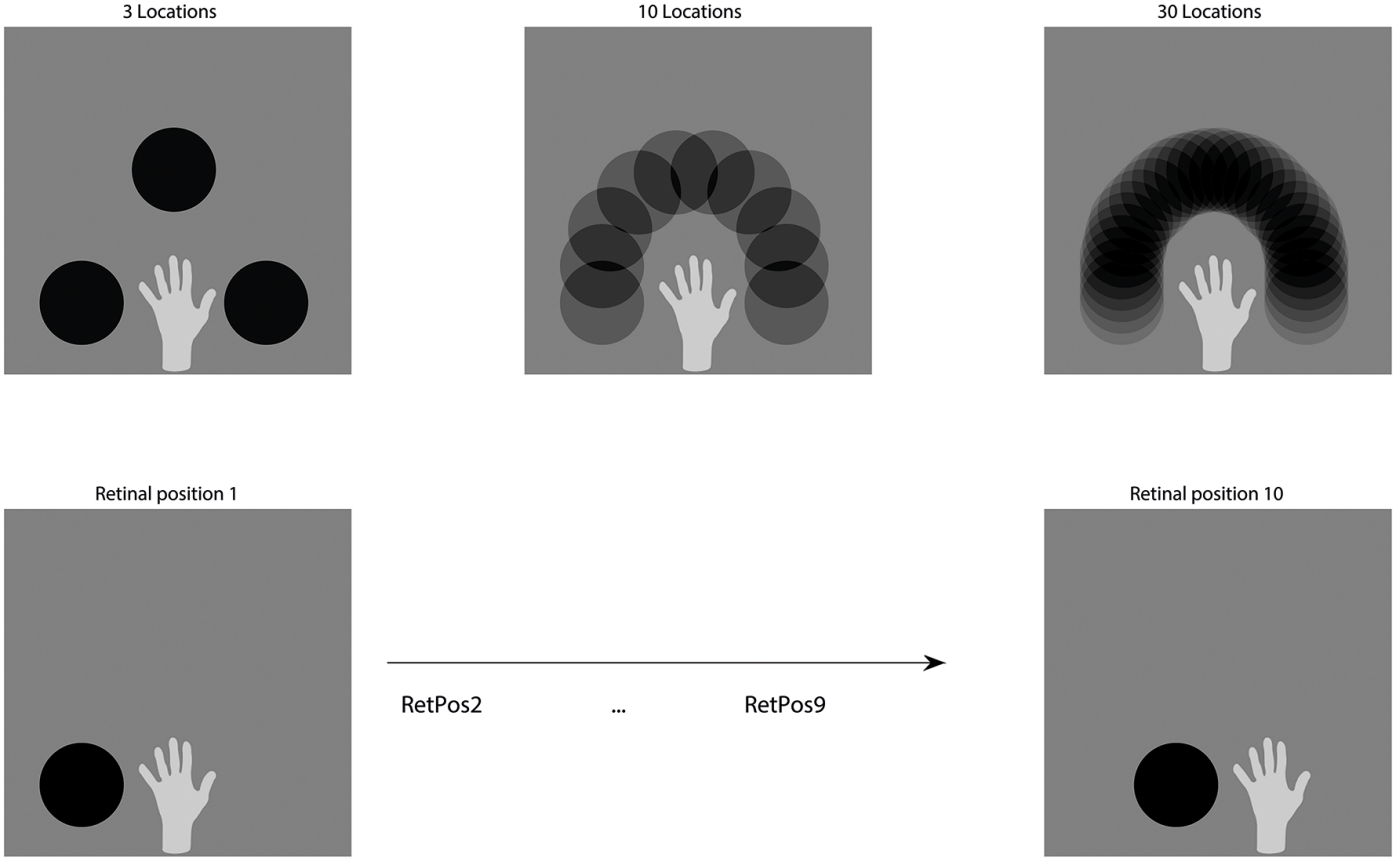
Training stimuli. The top row displays the relative arrangements between the target object and the hand (for all simulations) as well as how neighbouring target locations begin to overlap when the density of training locations increases—thus essentially forming a continuum of hand-centred locations. The brightness of the circle varies for visualization purposes. The bottom rows shows how a particular hand-object configuration was seen in different transforms during training by shifting it across the retina.

In section 3.1, we started with 3 object positions arranged equidistant on a semicircle around the hand and gradually increased the number of object positions to 10 by lowering the intermediate distances. Each of these hand-object configurations was shown in 10 different retinal positions during training, each shifted horizontally by 2 pixels, in order to simulate rapid saccades around the visual scene. To clarify, we present each hand-object configuration in all 10 retinal locations before moving on to the next hand-object configuration. Exemplary training stimuli are shown in the bottom row of Fig 3, while the upper row intends to clarify how the density of hand-centred locations was increased to effectively approximate a continuum.

In section 3.2 we evaluate the generalisation abilities of the hand-centred neurons by training exactly like in section 3.1, but testing the neurons on 20 highly overlapping hand-object configurations. In section 3.3 we check whether the order of presentation of the stimuli during training affects the development of hand-centred output neurons. In particular, in order to confirm that the network is not relying on a form of temporal binding to develop hand-centred neurons, we present all of the hand-object configurations in each retinal location before moving to the next retinal location. This training order corresponds approximately to a target object moving quickly through space while the hand remains at a fixed position. Since in this setup, the relative position between the hand and the target object varies rapidly rather than being stable over a short period of time, these control simulations rule out temporal binding as the mechanism producing hand-centred neurons (like demonstrated by Galeazzi et al.). To be precise, they aim to confirm the relative flexibility of CT learning as the underlying generative mechanism.

In section 3.4 we increase the number of hand-object configurations on which the network is trained to 15, 20 and 30 in order to investigate how well the output neurons learn to represent a near continuum of hand-centred target locations.

When presenting a particular image during training, the activations of individual neurons and their firing rates are calculated within a layer, and then the afferent synaptic connection weights are updated, as described in section 2.1. The presentation of all hand-object configurations across all 10 retinal locations constitutes one epoch of training. The network is trained one layer at a time, from layer 1 upwards to layer 4. For all the simulations described here, the network was trained for 40 epochs per layer.

## 3 Simulation results

### 3.1 Performance of network model when presented with discrete hand-object configurations

The aim of the initial simulations presented in this section was to confirm that CT learning could drive the development of hand-centred neurons in the output layer of VisNet, where such neurons would respond selectively to one particular hand-object configuration over all retinal locations. We began by running simulations in which the target object was presented at 3 equidistant hand-centred locations, ‘left’, ‘top’ and ‘right’. Then we gradually increased the number of hand-centred object locations to 10 by reducing the distance between neighbouring object locations. In each simulation, the hand-object configurations are presented with temporal continuity during training in a similar manner to that performed in the trace learning simulations of Galeazzi et al. [33]. That is, each hand-object configuration is shown shifting through all 10 retinal locations before moving on to the next hand-object configuration.

**Fig 4:**
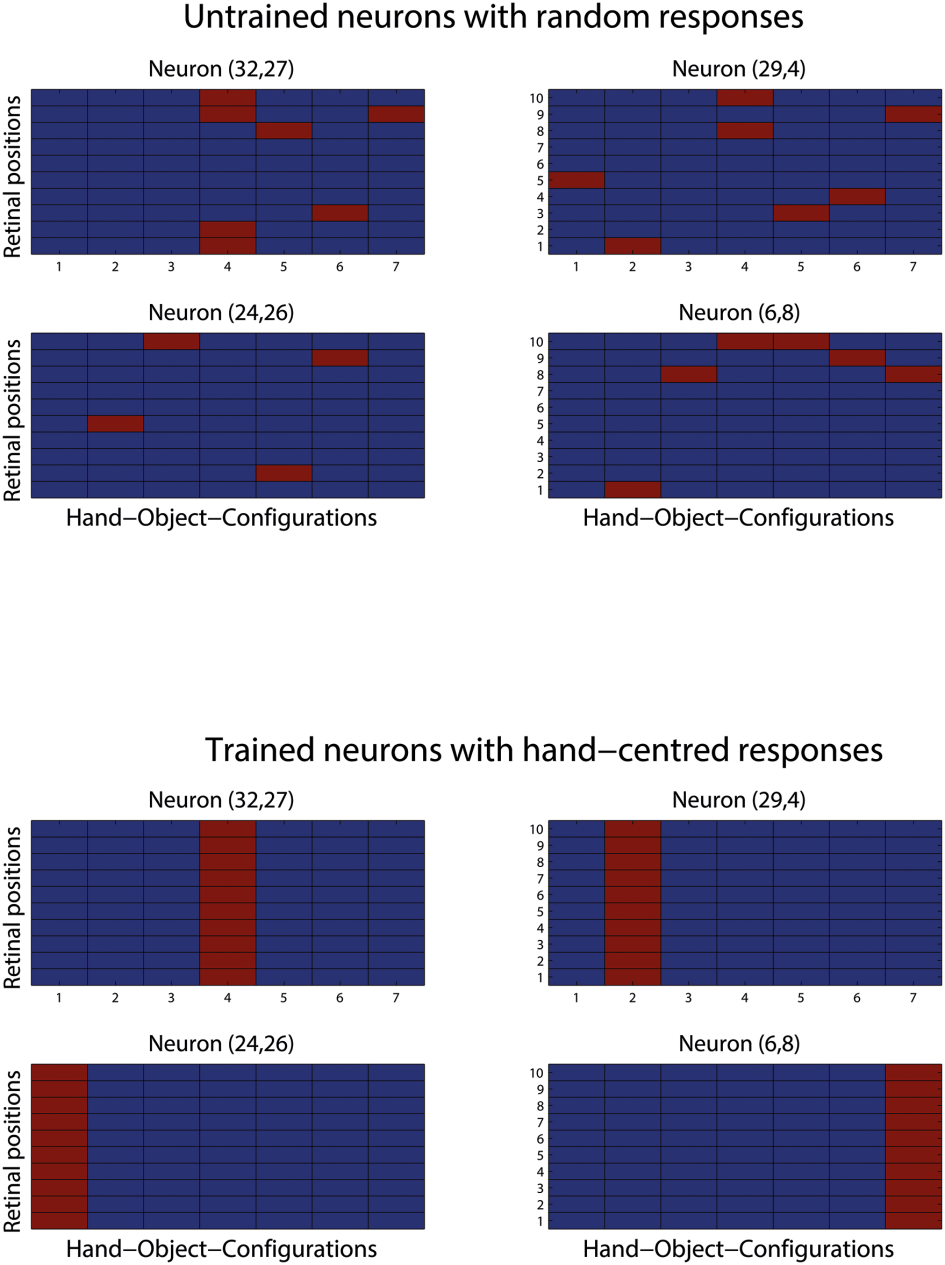
Response profiles of the same 4 output neurons before and after training in a simulation with 7 hand-object configurations. The top two rows show the binarised activation of 4 example output neurons before training. A red field indicates a response to a particular stimulus while a blue field indicates no response. The abscissa displays the 7 different relative configurations between hand and object, while the ordinate indicates all 10 retinal locations of each configuration. The bottom two rows show the firing rates of the same 4 neurons after training. Visual inspection reveals that before training the 4 neurons exhibit random firing when being presented with all 7 hand-object configurations across different retinal positions. However, after training, each of the 4 neurons responded to only one particular hand-object configuration, and responded to that configuration across all 10 retinal locations. These neurons had thus learned to display perfect hand-centred response characteristics.

Fig 4 shows the response profiles of 4 output neurons before and after training in a simulation with 7 hand-object configurations. Before training, the neurons responded randomly to the 7 hand-object configurations presented across different retinal positions. However, each of the same neurons learned to respond to a particular hand-object configuration across all retinal locations after training. For example, neuron (32, 27) learned to respond to the fourth hand-object configuration (i.e. the object is on top of the hand) and responded to this configuration across all 10 retinal locations. The 4 neurons shown in Fig 4 had thus developed perfect hand-centred firing properties after training.

**Fig 5:**
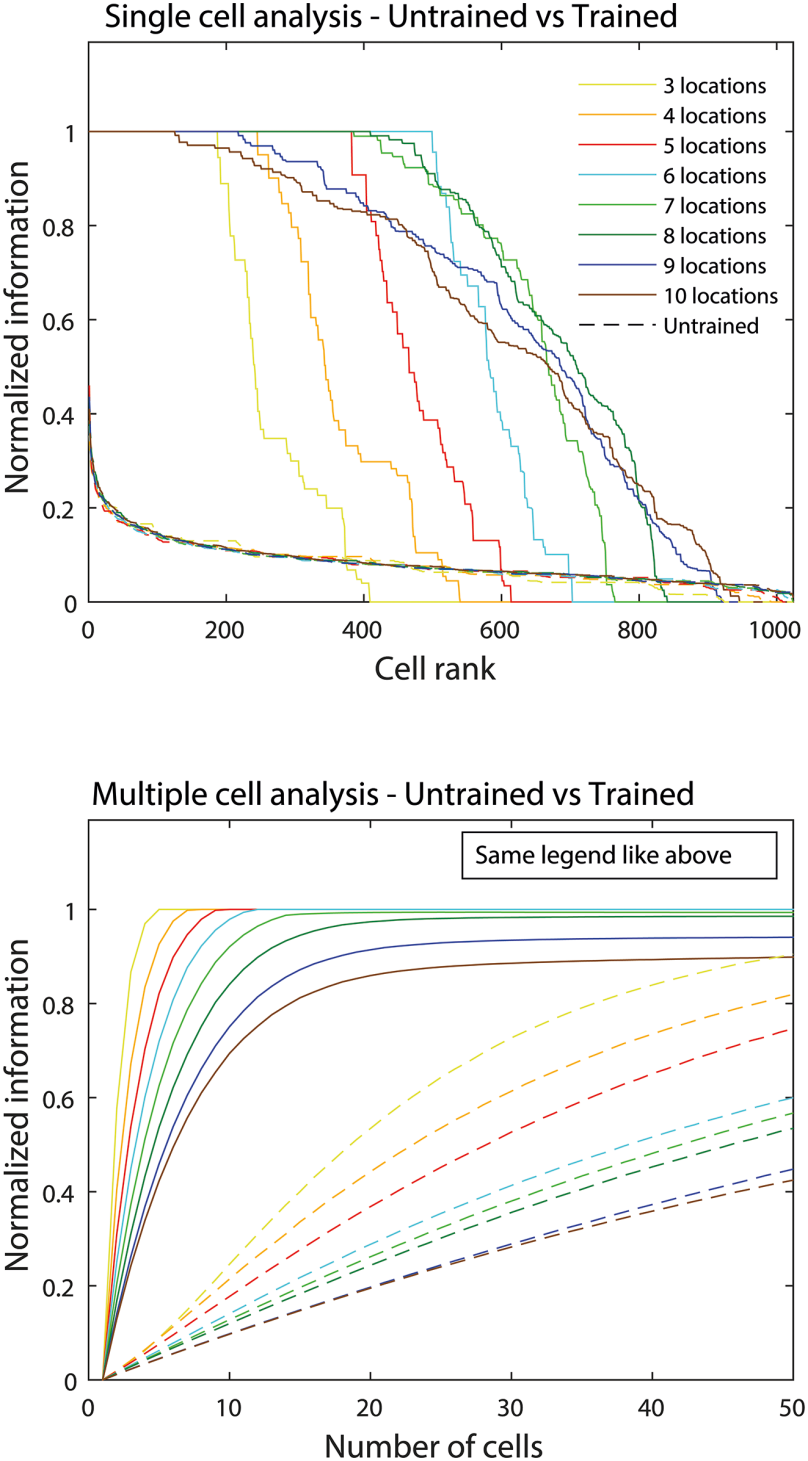
Single and multiple cell information measurements. The illustration shows the single and multiple cell information carried by neurons in the output layer for 8 separate simulations, each of which used a different number of hand-centred target locations ranging from 3 to 10. The upper plot compares the amount of single cell information carried by neurons in the output layer before training (dashed lines) and after training (regular lines) in ranked order. It can be seen that in all simulations training the network tuned at least 150 neurons to reach maximal single cell information after training, while there were no such neurons in the untrained condition. Visual inspection of the lower plot, which shows the multiple cell information for the same set of simulations, reveals that for all simulations training led to a large increase in the multiple cell information. In particular, for the simulations with up to 8 hand-centred target locations the multiple cell information approached the maximal value, which indicates that all hand-object configurations were represented by the output neurons.

For a more global assessment of network performance, Fig 5 presents the single and multiple cell information analyses for 8 separate simulations with different numbers of hand-centred target locations ranging from 3 to 10. The results shown for each simulation are in fact averaged over 10 simulations with different random seeds, as described in section 2.3.1. The upper plot of Fig 5 shows the single cell information carried by all 1024 neurons in the output layer for each simulation before and after training. For each simulation, the corresponding plots show the amount of single cell information carried by each neuron in ranked order. It is evident that, for all 8 simulations, the entire population of output neurons carried relatively little single cell information before training. However, in each of the 8 simulations, after training more than 150 neurons developed maximum single cell information. Since the maximal information varies by log_2_(Number of stimuli), the ordinate of Fig 5 is normalised to 1 for each simulation in order to facilitate comparison. Neurons with perfect hand-centred response characteristics, responded to just one hand-object configuration across all 10 trained retinal locations. We hypothesise that the higher number of hand-centred neurons for the case of 6 hand-centred target locations (531) compared with 3 target locations (212) results from the remaining representational capacity within our network. Furthermore, some drop off in performance could be seen in the simulations with the largest number (i.e. 9 or 10) of hand-centred target locations, with fewer neurons reaching the maximal level of single cell information in these simulations. In these cases, the hand-centred object locations are more tightly packed together and individual neurons may start to respond to two or more adjacent hand-centred object locations. However, this is entirely reasonable behaviour for a hand-centred neuron, since in the brain such neurons would in fact respond to small localised regions of hand-centred space. In other words, this last observation is a limitation of the current single cell information analysis, itself, which treats the hand-centred space as a finite number of discrete object locations rather than as a spatial continuum. It is for this reason that additional analytical methods were developed above in section 2.3.2 to treat the hand-centred space as a continuum, and which are applied to simulation data below in section 3.2.

The lower plot of Fig 5 shows the multiple cell information for the same set of 8 simulations with different numbers of hand-centred object locations. For each simulation, the multiple cell information was computed across the 5 cells with maximal single cell information for each hand-object configuration. It can be seen that there was a large increase in the multiple cell information carried by output neurons after training. Moreover, in simulations with up to 8 target object locations, the multiple cell information asymptoted to the theoretical maximal value. This indicated that all of the different hand-object configurations were properly represented by the output neurons in these simulations. However, as seen with the single cell information analysis, there was an apparent drop in the asymptotic value of the multiple cell information for those simulations with the largest number (i.e. 9 or 10) object locations. Again, this is to be expected as the density of hand-centred object locations is increased, resulting in individual hand-centred neurons learning to respond to a small cluster of nearby hand-centred object locations.

In summary, these simulation results confirm our hypothesis that CT learning, which relies on a purely Hebbian learning rule without a memory trace of recent neuronal activity as used in the trace learning model of Galeazzi et al. [33], could drive the visually-guided development of hand-centred visual neurons. Such neurons respond selectively to a target object in a particular location with respect to the hand, and continue to respond to that configuration invariantly as it is presented across different retinal locations. The simulations described above demonstrate for the first time that these hand-centred cell response properties can develop through a biologically plausible, Hebbian learning mechanism.

### 3.2 Ability of network model to generalise to novel stimulus-object configurations

In the simulations presented in the previous section, the network was trained and tested on the same set of hand-object configurations. However, the visual brain must be able to represent a spatial continuum of target object locations with respect to the hand. Can our model represent such a continuum of hand-centred locations after training on only a limited number of discrete hand-object configurations? In this section, we address this question by exploring the ability of the model to generalise its responses to a (near) continuum of mostly novel hand-centred target locations not encountered during training.

In each of the simulations presented in this section, the network was trained on a discrete number of hand-object configurations, ranging from 3 to 10 configurations, as presented above in section 3.1. However, in contrast to the previous section, testing was done with an increased density of 20 highly overlapping hand-object configurations that were presented across different retinal locations. This meant that the model was being tested on a (near) continuum of hand-centred target locations, most of which had not been seen by the network during training. For example, presenting the object at 10 equidistant positions around the hand corresponded to an angle of 22° between neighbouring positions, while the angle is 10.6° respectively 6.9° for 20 and 30 locations. Accordingly, training on 10, but testing on 20 locations implies that the position of every second tested location to the closest known, training location is maximized; which clearly hampers task difficulty. Therefore, if hand-centred neurons are successfully shown to generalize to the object presented at the precise middle between two trained locations, then they will also generalize to all intermediate positions, which supersede the high computational effort of testing on a denser continuum of locations dispensable.

The novel hand-centred target locations represented intermediate positions between the trained hand-centred target locations, like shown in Fig 3.

As the density of *tested* hand-centred target locations effectively approaches a continuum, we need to verify whether each output neuron learns to respond to a localised region or cluster of hand-centred locations, rather than responding to just a single hand-object configuration. Consequently, in this section we begin to employ the more flexible criterion described above in section 2.3.2 for assessing whether output neurons develop hand-centred responses. In this case, a neuron is classified as hand-centred if it responds to a small, localised cluster of hand-centred target locations across a large number of retinal locations.

It is also important to check that the receptive fields of the entire population of hand-centred output neurons are distributed reasonably evenly across the 20 hand-centred target locations on which the network is tested. If we observe approximately the same number of hand-centred neurons selective to each of the 20 hand-centred target test locations, then this will imply that the trained model provides an adequate visual representation of the whole hand-centred space, and that the population of hand-centred neurons maintains a similar overall level of activity for objects shown in different locations with respect to the hand.

Consequently, from the population of output neurons that were classified as hand-centred according to the criterion described in section 2.3.2, we calculated the number of hand-centred neurons that represented each of the (tested) target locations with respect to the hand. This was achieved in 2 successive steps as follows. In step 1, we recorded the number of times that each hand-centred output neuron *i* responded to each hand-object configuration *j* when that configuration was presented over all ten retinal positions, and this number of responses is denoted by $R_{j}^{i}$. In step 2, for each hand-centred output neuron *i* we added a contribution of $\frac{R_{j}^{i}}{\sum_{k=1,N}R_{k}^{i}}$ to the *j*th value of the frequency distribution for each hand-object configuration *j*. Given this procedure, summing over all the final frequency values in the distribution gives the total number of hand-centred neurons.

**Fig 6:**
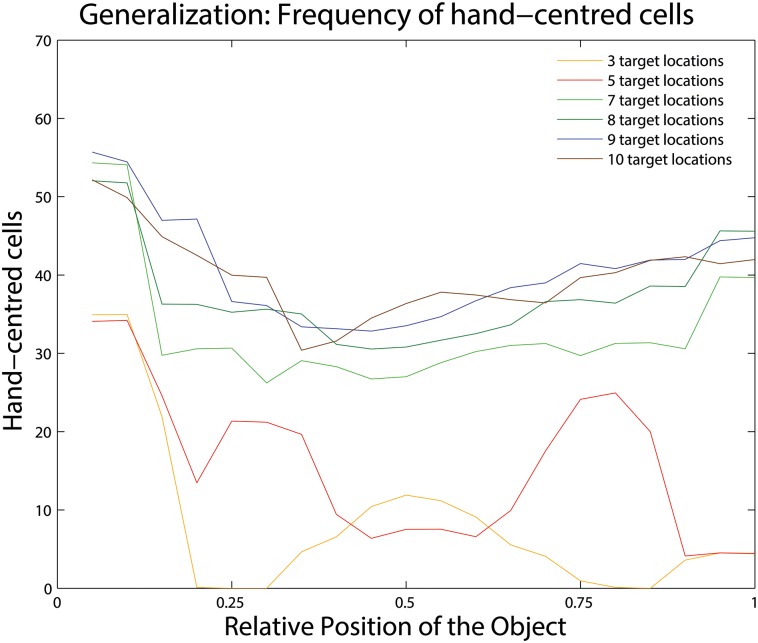
The ability of output neurons trained on a limited number of discrete hand-centred locations to generalise their responses to a spatial continuum of hand-centred locations. Results are shown for 6 separate simulations in which the network was trained on *N* = 3, 5, 7, 8, 9, 10 hand-centred target locations, but tested on a (near) continuum of 20 highly overlapping hand-centred target locations. The figure shows how the receptive fields of the population of hand-centred output neurons are distributed across the 20 hand-centred target locations used to test the network. That is, for each simulation we plot the number of hand-centred neurons assigned to each of the 20 test locations, where, on the abscissa, 0 denotes the target object at the leftmost hand-centred position and 1 at the rightmost position. It can be seen that for all simulations in which the network was trained with 7 or more hand-centred target locations, the network developed hundreds of hand-centred output neurons. Moreover, these hand-centred output neurons were distributed fairly evenly across the 20 hand-centred test locations. These results confirm that, for simulations in which the network is trained with only a limited but sufficient number (i.e. 7) of hand-centred target locations, the hand-centred output neurons are able to generalise their responses to a spatial continuum of hand-centred locations.

Based on this computational procedure, Fig 6 shows the number of hand-centred output neurons with receptive fields localised at different test positions across the hand-centred space. Results are shown for 6 separate simulations in which the network was trained on *N* = 3, 5, 7, 8, 9, 10 hand-centred target locations, but tested on 20 highly overlapping hand-centred target locations. The results shown are, in fact, averaged across 10 different simulations using altered random seeds to initialise the network connectivity. It is evident that for those simulations in which the network was trained with 7 or more hand-centred target locations, the network developed many (i.e. hundreds) of hand-centred output neurons as classified according to the criterion described in section 2.3.2. Furthermore, and most importantly, the receptive fields of the hand-centred output neurons were distributed reasonably evenly across the 20 hand-centred locations used to test the network. These results confirm that, for simulations in which the network is trained with only a finite number (i.e. 7, 8, 9 or 10) of discrete hand-centred target locations, the population of hand-centred output neurons is able to generalise its neuronal responses to, and thereby adequately represent, a spatial continuum of hand-centred locations.

### 3.3 Effect of temporal interleaving of different hand-object configurations during training

A key predicted property of the proposed CT learning mechanism for the development of hand centred visual neurons is that CT learning should not require that each spatial configuration of the hand and a target object is seen with temporal continuity as it shifts across the retina. For example, CT learning should still be able to bind together images of the same hand-object configuration seen in different retinal locations even if different hand-object configurations are seen rapidly interleaved with each other through time. This important property, which sharply differentiates the CT learning mechanism proposed in this paper from the trace learning approach of Galeazzi et al. [33], is now tested in this section.

**Fig 7:**
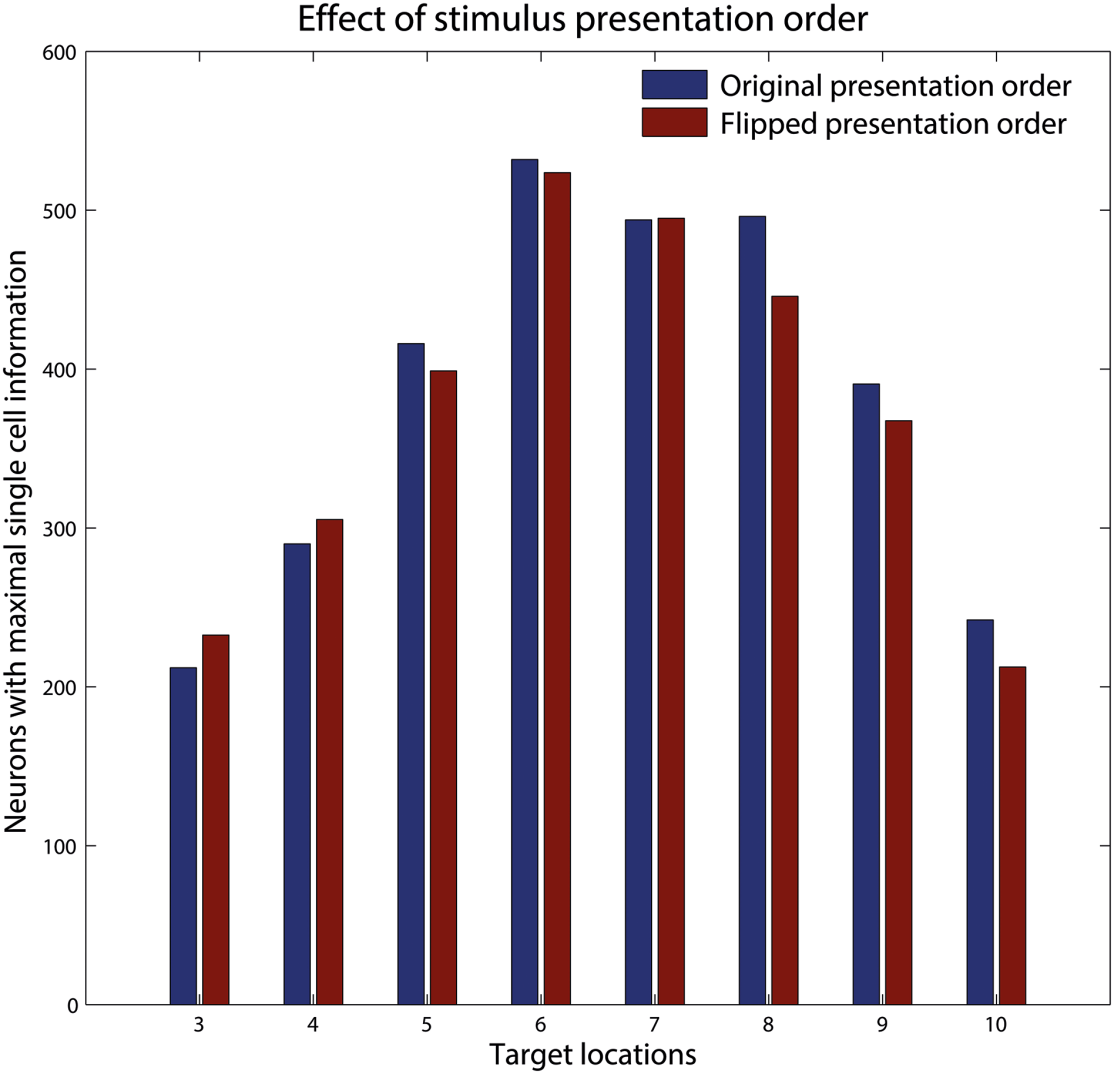
Effect of stimulus presentation order. Comparison of simulation results obtained when the stimuli are presented in two different temporal orders during training. In the first set of simulations (blue) the stimuli are presented in the original order used in section 3.1, while in the second set of simulations (red) the order of the stimulus presentations is flipped. For each of these two training conditions, we compare the average number of output neurons that achieved perfect hand-centred firing characteristic when using a different number of hand-centred target locations ranging from 3 to 10. It can be seen that there is no substantial difference in the numbers of neurons developing hand-centred responses with the two different training conditions. This confirms that the CT learning mechanism operates very robustly with respect to changes in the presentation order of stimuli during training.

In both previous sections, each hand-object configuration was shown shifting through all 10 retinal locations before moving on to the next hand-object configuration. For the new simulations carried out in this section, we used the same set of training stimuli as used above in section 3.1. However, now we flipped the presentation order of stimuli during training. Specifically, in this next set of simulations all of the different hand-object configurations are shown at a particular retinal location before moving to the next retinal location. This led to a rapid temporal interleaving of different hand-object configurations during training such that not any two consecutively presented training stimuli revealed the same hand-object configuration. Given our supposition that CT learning should not be sensitive to the temporal ordering of stimulus presentation, we predicted that the new temporal ordering of stimulus presentation used in this section should give similar results to that obtained with the original stimulus presentation order used in section 3.1. Fig 7 compares simulation results obtained when the stimuli are presented in either the original presentation order used in section 3.1 (results shown in blue), in which each hand-object configuration is shown shifting through all 10 retinal locations before moving on to the next hand-object configuration, or presented using the new flipped order (shown in red). For each of these two different stimulus presentation orders, the figure shows the average number of perfectly hand-centred neurons in the output layer (carrying the maximum level of single cell information) for 8 simulations with different numbers of hand-centred configurations ranging from 3 to 10.

It is evident from the figure that similar numbers of neurons developed perfect hand-centred response characteristics with the two alternative training conditions. We confirmed this observation by conducting *one-tailed t-tests* for the 8 separate sets of simulation results with 3 to 10 hand-centred object positions, none of which revealed a significant difference in the results obtained with the two training conditions. In order to get a single, global measure comparing the outcomes of all 8 sets of simulations, a *two-sided Wilcoxon signed-rank test* was used and returned a *p*-value of 0.511.

The results described above confirm that the new CT learning mechanism proposed in this paper provides very robust performance with respect to variation in the presentation order of alternative hand-object configurations in different retinal positions during training. This robustness is a known property of CT learning, originally reported in the development of transformation invariant representations of objects in the ventral visual system. In particular, it is demonstrated that CT learning does not require that different retinal views of the same hand-object configuration are seen clustered together in time. Thus, CT learning enables the development of hand-centred neurons by binding together different retinal views of the same hand-object configuration on the basis of their spatial similarity or overlap, as described in section 2.2.

### 3.4 Performance of the model when trained with a continuum of hand-centred target locations

In all the simulations described above, our model was trained with at most 10 different hand-object configurations. However, the relative configurations of the hand and nearby objects that humans see during everyday experience is much higher, approaching a continuum of object locations with respect to the hand. Consequently, in this section, we investigate whether our model is able to successfully develop hand-centred output neurons when the network is trained with a (near) continuum of hand-centred object locations. To this end, we conducted simulations with 15, 20 or 30 highly overlapping, equidistant hand-centred object locations arranged in a circular arc around the hand. In these cases, rather than learning to respond to only a single hand-object configuration, each output neuron should learn to respond to a localised region or cluster of hand-centred locations, like in section 3.2. Furthermore, the receptive fields of the population of hand-centred neurons should be evenly distributed throughout the space around the hand in order to provide an adequate visual representation of the whole space. Investigating the performance of our model on these more ecologically realistic training conditions, in which the hand-centred target locations form a (near) spatial continuum around the hand, denotes a crucial step in verifying the plausibility of CT learning as a mechanism that could drive the development of hand-centred neurons in the brain.

**Fig 8:**
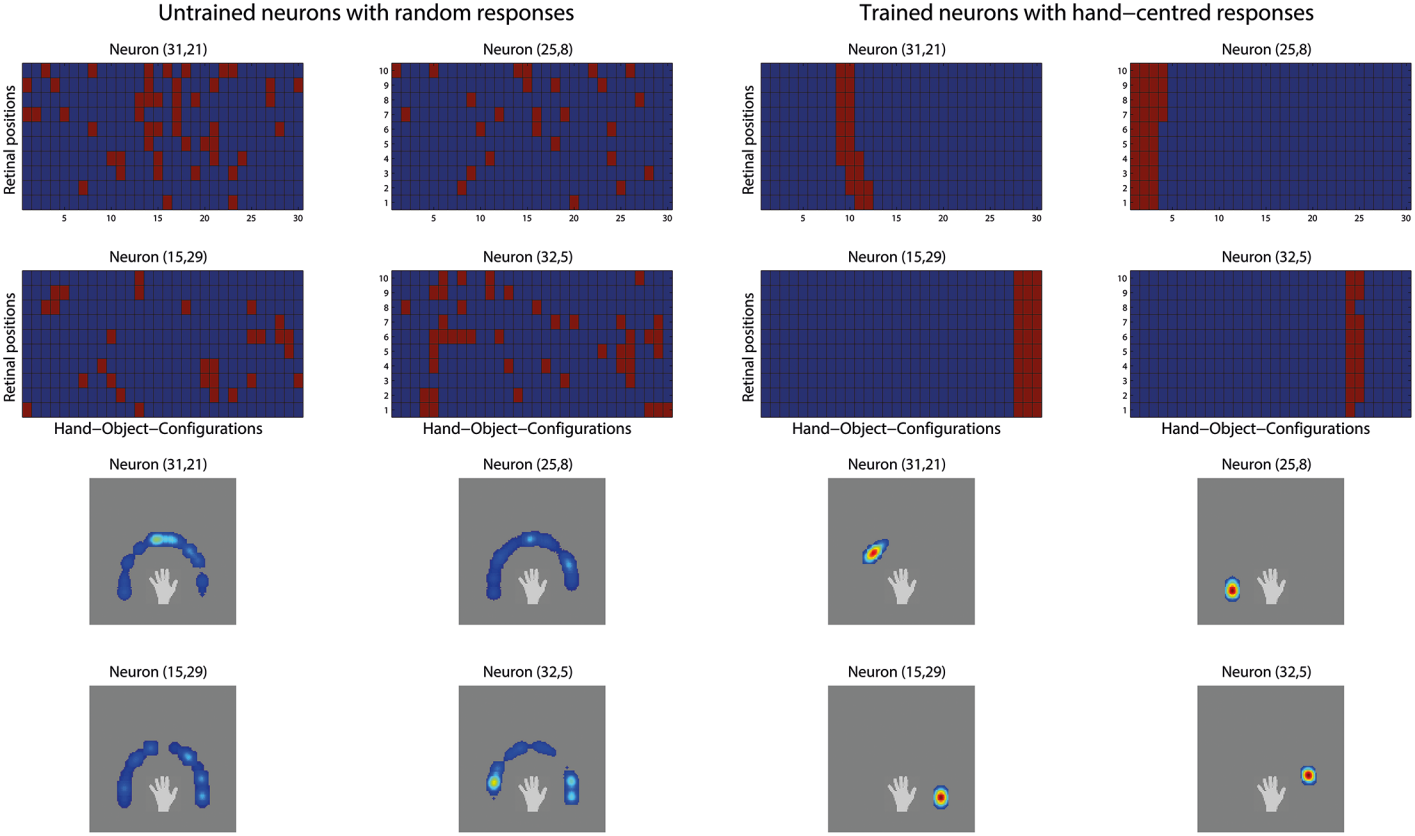
Response profiles of 4 output neurons when trained on a continuum of hand-centred target locations. In this simulation, the network was trained on 30 highly overlapping hand-object configurations in order to simulate a (near) continuum of target locations in a circular arc around the hand. The two left columns of this figure show the responses of 4 example neurons in the output layer before training. The two upper rows show the binary response matrices of the 4 neurons to the complete set of 30 hand-object configurations presented across all 10 retinal positions. The two lower rows present the same data, where the responses for each neuron have been averaged over the 10 retinal positions and then plotted with respect to the hand. The two right columns display the firing behavior of the same neurons following the same conventions but after training. It can be seen that before training the neurons initially responded randomly to different hand-object configurations presented in different retinal positions. However, after training, each neuron had learned to respond to a localised cluster of hand-centred target object locations, and responded to this localised region of hand-centred space across different retinal locations.

As the density of hand-centred target locations effectively approaches a continuum, we verified whether each output neuron learns to respond to a localised region or cluster of hand-centred locations—like in section 3.2. Fig 8 shows the binarised firing responses of 4 example output neurons before and after the network was trained on 30 hand-centred target object locations presented across 10 retinal locations.

Before training, the neurons initially responded randomly to the different hand-object configurations presented in different retinal positions. This can be seen from the scattered red fields within the binary response matrices shown in the top rows of the first two columns. Again, a red field indicates a response while a blue field indicates no response. The behaviour of the same neurons after training can be seen in the two right columns. It is evident that, after training, each of the 4 neurons developed sensitivity to a localised cluster of hand-centred target object locations, and responded to their preferred localised region of hand-centred space across different retinal locations. For example, neuron (32, 5) learned to respond when the object is presented at hand-centred positions 24 or 25 and responded to almost all shown retinal locations. As the response plots show, each of the 4 output neurons developed clearly localized, hand-centred receptive fields: responding to a short range of similar hand-object configurations almost completely irrespective of where this stimulus was presented on the retina. A theoretical explanation why training on a continuum of target locations still lead to the development of hand-centred neurons, rather than the undesired, alternative supposition of responding selectively to retinal locations, is presented in the next section.

**Fig 9:**
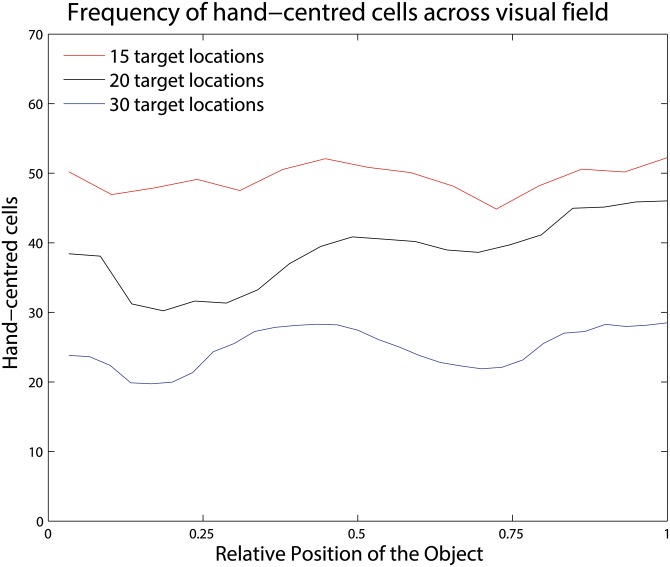
Frequency of hand-centred neurons with receptive fields localised at different positions across the hand-centred space. The illustration shows results for three separate simulations in which the network was trained with *N* = 15, 20 or 30 hand-centred target locations. The conventions used in this figure are the same like in Fig 6. It can be seen that the distribution of hand-centred neurons across the hand-centred space is approximately uniform for simulations with 15, 20 or 30 hand-centred target locations. These results confirm that CT learning produced an even visual representation of the space around the hand for all three simulations.

As discussed above, a key question is whether the receptive fields of the population of hand-centred neurons that develop during training are evenly distributed through the space around the hand, and whether these hand-centred neurons cover the entire hand-centred visual space and successfully represent all trained hand-object configurations. Following the procedure in Figs 6 and 9 shows the number of hand-centred neurons with receptive fields localised at different positions across the hand-centred space scaled to the range [0, 1].

Results are shown for 3 separate simulations in which the network was trained with 15, 20 or 30 hand-centred target locations. The plots for all three simulations show that the distribution of hand-centred neurons across the space around the hand is approximately uniform. As a further quantitative test, the standard deviations of the three plotted distributions were found to be in the range of only 5-12% of the mean values. These results confirm that the purely Hebbian CT learning mechanism produced approximately the same number of neurons representing each of the trained hand-object configurations, and hence an even visual representation of the space around the hand, for all 3 simulations.

### 3.5 A theoretical explanation for the development of hand-centred neurons

The simulation results presented above in section 3.4 confirmed that when the network model is trained on a (near) continuum of hand-centred target locations, it continued to develop hand-centred output neurons with small localised receptive fields in hand-centred space. However, this leaves open the major theoretical question why the network developed such hand-centred output neurons rather than producing individual neurons that respond to many different spatially overlapping hand-object configurations at the same retinal location.

The underlying problem is that when the network is presented with a continuum of highly overlapping hand-centred target locations, CT learning could potentially operate in alternative ways as follows. Firstly, CT learning could continue to drive output neurons to bind together small subsets of highly similar hand-object configurations presented across different retinal locations, and thereby produce hand-centred neurons with localised hand-centred receptive fields. Alternatively, CT learning could drive output neurons to bind together many different hand-centred target locations at a particular retinal location due to their spatial overlap, and hence produce output neurons that respond to many different hand-object configurations at that retinal position. This would correspond to horizontal rather than vertical red bars in Fig 8. Consider, that both suppositions are in accordance with the CT learning hypothesis (2.2). Finally, CT learning could combine these two tendencies in an unconstrained and random manner, thus producing output neurons with highly erratic response characteristics. However, in practice, the simulations reported above in section 3.4 confirmed the continued development of perfect hand-centred neurons, even as the number of hand-centred target locations was increased to 100. In this section, we try to develop a deeper theoretical understanding of these important simulation findings. Specifically, we hypothesise that these simulation findings may be understood by considering a Principal Component Analysis (PCA) of the underlying sources of variance in the visual training stimuli consisting of alternative hand-object configurations presented across different locations on the retina.

The VisNet model used in the simulations presented in this paper consists of a hierarchy of competitive neural layers. Competitive networks are frequently used as unsupervised pattern classification algorithms due to their ability to cluster together similar input patterns while separating dissimilar input patterns. In particular, they are able to produce far more compressed representations of the input patterns by removing redundancies in these representations. Within each layer, the excitatory neurons receive afferent synaptic connections from a topologically corresponding, localised region of neurons within the preceding layer. These bottom-up synaptic connections self-organise during visual training using the Hebbian learning rule Eq (7) followed by renormalisation of each neuron’s synaptic weight vector at the end of each timestep. This learning process bears some comparison with the operation of Oja’s learning rule [51], which may itself be derived from the operation of Hebbian learning with weight vector renormalisation and also bounds the length of the converged synaptic weight vector. Interestingly, Oja’s rule when applied to a single neuron has been shown to extract the first principal component of the input training data. In later work, Sanger [52] chained together Oja’s neurons allowing for a full Principal Component Analysis (PCA) and called this technique the Generalized Hebbian Algorithm.

We speculated that the neurons in the competitive output layer of VisNet may be carrying out a somewhat similar PCA operation. That is, individual output neurons may learn to represent input patterns with some correspondence to the largest sources of variation over the entire stimulus set used to train the network. The stimulus set used for each of our simulations consists of a number of different hand-object configurations shown across all retinal locations. We hypothesised that the greatest source of variation within the stimulus sets was due to changes in the position of target objects with respect to the hand rather than changes in the retinal location of hand-object configurations. In this case, applying PCA to such a stimulus set should lead to the highest principal components representing the hand-centred positions of target objects, with the retinal position of hand-object configurations represented by lower principal components. Most importantly, if the output neurons in our model are indeed learning to represent the directions of highest variance, then the lateral inhibition will drive them to be sensitive to the hand-centred locations of objects rather than the location of hand-object configurations on the retina. So, do the first eigenimages of those stimulus sets that were successfully used to develop hand-centred output neurons, represent the locations of target objects with respect to the hand? In order to investigate this, we applied PCA to the stimulus sets of 6 simulations. These simulations varied according to the number of hand-centred target locations, number of retinal locations of each hand-object configuration, and the size of the retinal shifts of hand-object configurations (in pixel), with these simulation parameters listed in Table 4.

**Table 4:** Parameters of the training image sets used for the simulations represented in Fig 10. The stimulus sets used for the PCA were parameterized according to the rows (a) to (f). Each set consisted of images of hand-object configurations shown across different retinal locations.

	Target locations	Retinal shifts	Size of shifts
a	4	10	2
b	30	10	2
c	4	10 × 10	2
d	4	10	5
e	4	4	5
f	4	25	2

**Fig 10:**
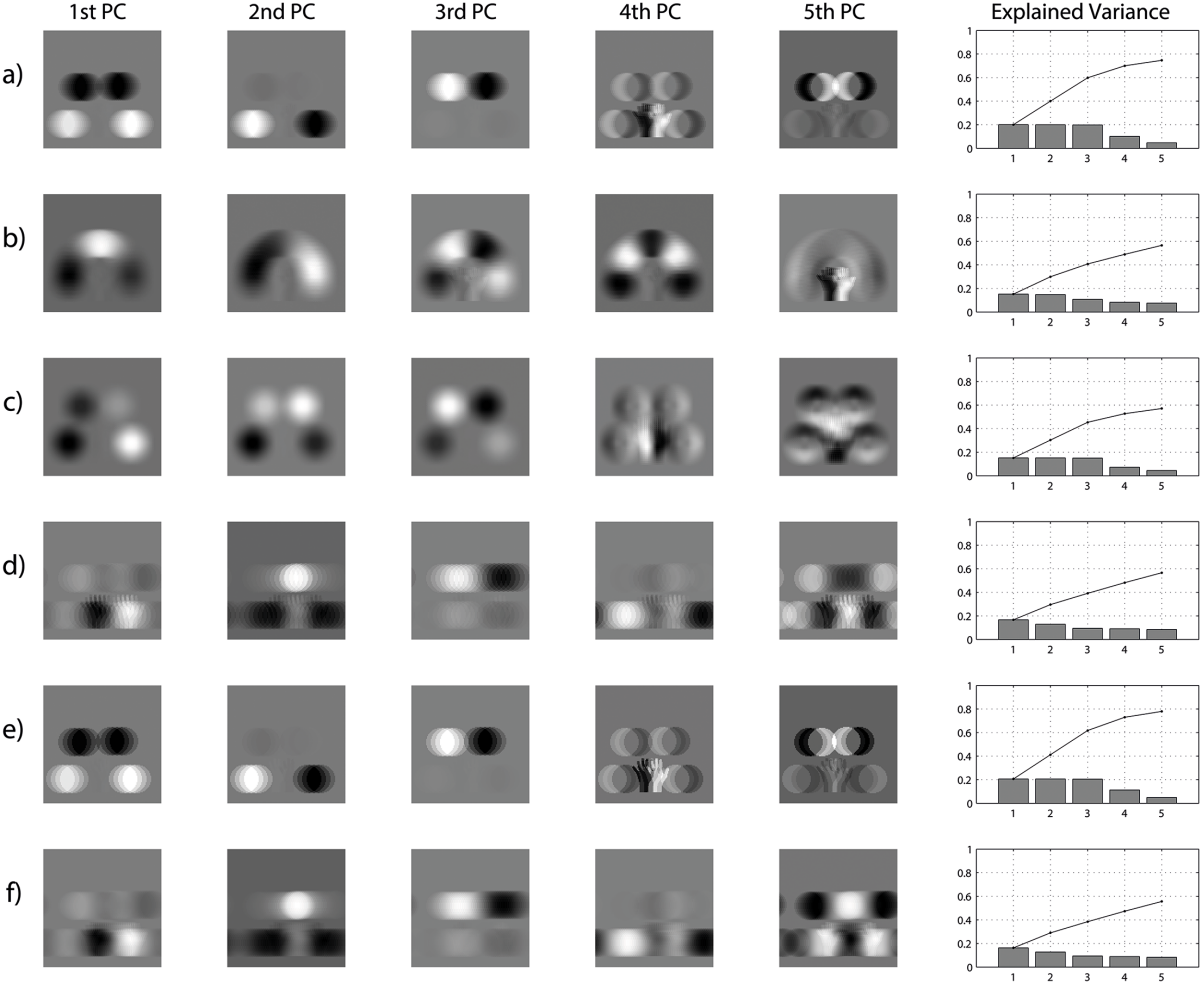
Eigenimage representations of stimulus sets used for six selected simulations. The results of a PCA of the stimulus sets used for six different simulations are shown in rows (a) to (f) respectively. The stimulus set for each simulation consists of images of a number of alternative hand-object configurations presented across all retinal locations. These sets were parameterized according to Table 4. Columns 1 to 5 show the first five eigenimages of the stimulus set used for the simulation represented in each row. The sixth column shows the (cumulative) explained variance of the first 5 principal components. The simulations represented in rows (a), (b), (c) and (e) developed hand-centred output neurons, while the simulations shown in rows (d) and (f) did not develop hand-centred neurons. It is evident from the eigenimages shown in the figure that for those simulations that produced hand-centred output neurons, the highest principal component eigenimages represent the positions of targets with respect to the hand rather than the retinal location of hand-object configurations. Thus, for those simulations in which hand-centred output neurons developed, the greatest source of variance arises from changes in the hand-centred locations of target objects rather than changes in retinal locations of the hand-object configurations.

The eigenimages presented in Fig 10 resulted from applying PCA to the stimulus sets used for these simulations. This representation revealed the greatest sources of underlying variance in each stimulus set in descending order. The simulations represented in rows (a), (b), (c) and (e) developed hand-centred output neurons, while the simulations shown in rows (d) and (f) did not develop hand-centred neurons. From Fig 10 it can be seen that for those simulations that developed hand-centred output neurons, the first eigenimages represented the locations of targets with respect to the hand, rather than the location of the hand-object configuration on the retina.

This effect is particularly evident for the simulation shown in row (a) of Fig 10, for example. Here we can see that the first three eigenimages differentiate between different hand-centred positions of the target object. In row (a) we can see two light regions (signifying higher values) in the bottom left and bottom right of the eigenimage, where each of these light regions corresponds to a circular target object presented in a particular location with respect to the hand and translated horizontally across a number of retinal locations. Similarly, two dark regions (signifying lower values) can be seen in the centre left and centre right of the eigenimage, where each of these dark regions also corresponds to a circular target object presented in a particular hand-centred location and translated horizontally across a number of retinal locations. Importantly, though, there is no image of the hand present within the first eigenimage. Consequently, this eigenimage, which represents the greatest source of variance in the stimulus set, separates the upper from lower hand-centred object positions. In a similar manner, the second principal component in row (a) distinguishes between the leftmost and rightmost hand-centred object positions. Most importantly, this is independent of the retinal location of the hand-object configuration since the horizontal shifts of the hand are represented by the fourth principal component with a clear drop in the corresponding eigenvalue shown in the sixth column. Thus, the highest principal components for the simulation represented in row (a) clearly reflect the variance due to changes in target location with respect to the hand. Similar trends are also seen for the simulations represented in rows (b), (c) and (e). For example, the stimulus set for the simulation shown in row (b) contains a (near) continuum of 30 hand-centred target locations, and yet still develops hand-centred output neurons after training (see section 3.4. In row (b) we can see that the first four principal components differentiate between different hand-centred target locations, while the fifth principal component represents horizontal shifts of the hand. Thus, for those simulations that developed hand-centred output neurons, the PCA confirms that the greatest source of variance arises from changes in the hand-centred locations of target objects rather than changes in retinal locations of the hand or hand-object configurations.

In contrast, the simulations that did not develop a single perfect hand-centred output neuron are represented in rows (d) and (f) of Fig 10. For these simulations the hand-object configurations were presented shifting across a relatively large horizontal region of retinal space, where each hand-object configuration was presented across a grid of 50 pixels that was covered by 10 shifts of 5 pixels and 25 shifts of 2 pixels, respectively. With these settings, the variance in the stimulus set due to translation of hand-object configurations across the retina is highest. Consequently, it can be seen that the first eigenimages shown in rows (d) and (f) represents horizontal shifts of the hand. To further clarify our interpretation of these eigenimages, note that the first principal components in rows (d) and (f) each contain a pair of light and dark images of the hand in the central bottom region of the eigenimage, where the light and dark hand images occupy different retinal locations on the right and left, respectively. These eigenimages thus separate different retinal positions of the hand. Hence, for those simulations that did not develop hand-centred output neurons, the greatest source of variance arises from changes in retinal locations of the hand or hand-object configurations. The simulation represented in row (c), in which the hand-object configurations were shifted across a 10 × 10 grid rather than only horizontally as in other rows, still developed a large number of hand-centred output neurons. We hypothesize that this results from extending the underlying source of variance in the stimulus set by another orthogonal (vertical) dimension rather than increasing the variance in the already existing (horizontal) dimension. This explanation is suggested by the 4th and 5th eigenimages of row (c), which represent shifts of the hand in the two orthogonal directions.

The PCA results shown in Fig 10 provide strong support for the hypothesis that it is the underlying sources of variance in the stimulus set that is driving self-organisation within the network when relying on the Hebbian learning rule with renormalisation of synaptic weight vectors at each timestep. In particular, the output neurons develop hand-centred responses when the greatest source of variance in the stimulus set, as may be revealed by PCA, is due to changes in the positions of target objects with respect to the hand.

In conclusion, based on the above findings, we hypothesise that the output neurons in the network model are extracting and representing the dimensions of largest variance within the input data, which in these simulations is due to variation in the position of target objects with respect to the hand. Further, lateral inhibition between the output neurons, which introduces competition between the neurons, forces individual neurons to learn to respond to different parts along the dimensions of highest variance. Indeed, this corresponds to particular hand-object configurations. Moreover, CT learning, which binds together subsets of input patterns based on their spatial overlap or similarity, is encouraging output neurons to bind together input patterns along the dimension of least variance, that is, binding together particular hand-object configurations across different retinal locations. In this case, output neurons learn to respond selectively to specific hand-object configurations, or rather small localised regions of the space of hand-centred object locations when dealing with a spatial continuum, irrespective of retinal location. In other words, retinal locations are bound together for particular hand-object configurations rather than vice versa, inducing the development of output neurons that fire in a hand-centred frame of reference.

## 4 Discussion

In this paper we have presented the first approach to utilise an invariance learning mechanism known as Continuous Transformation learning [37] to drive the development of hand-centred output neurons that respond invariantly over different retinal locations in a biologically plausible, hierarchical neural network model of the primate visual system. The CT learning mechanism operates by encouraging output neurons to bind together input patterns due to their spatial overlap or similarity, and in this way can train individual neurons to respond to particular hand-object configurations across the retina. CT learning as an explanation for the development of hand-centred visual neurons has a couple of advantages over a previous model developed by Galeazzi [33], which utilised trace learning rather than CT learning. Firstly, CT learning operates with a simpler, purely Hebbian form of learning rule which does not need to incorporate a memory trace of recent neuronal activity. Secondly, and more importantly, CT learning does not require that different retinal views of the same hand-object configuration are seen clustered together in time. Instead, CT learning is robust enough to cope with a much broader and more natural range of visual training sequences as simulated in section 3.3. This includes conditions in which the relative positions of the hand and nearby objects are changing rapidly or are static over a long period of time. In a nutshell, our simulation results provide the first evidence validating CT learning as a potential mechanism underlying the development of hand-centred neurons in the primate brain. Furthermore, the results specify and formalise the work of Haykin [53], who has claimed that an early benchmark model of the visual system developed by Linsker [54], a hierarchical network with topological bottom-up synaptic connectivity comparable to VisNet, learned to discriminate localised regions along the directions of highest information content (i.e. maximum variance) within the input data. In addition, both CT learning and PCA are independent of the temporal order in which the training stimuli are presented to the network, which accounts for the observation in section 3.3 that the temporal ordering of the training stimuli has no significant effect on the development of hand-centred output neurons in the network.

In this paper, our goal was to visually tune neurons to fire in a specific frame of reference (which may be interpreted as a specific type of learning translation invariance). While translation invariance arises inherently in some supervised neural network architectures, such as convolutional neural networks (due to the shared weights), it needs to be pointed out that translation invariance is an inherently difficult task for self-organizing, competitive networks compared to e.g. learning rotation invariance. The latter case follows conventional principles of competitive learning: a cluster of overlapping input patterns is associated together by a weight vector pointing towards the centre of that cluster [37, 40]. This assumption of high correlation between different transforms cannot be made for translation invariance tasks where an object might occur at such broad positions in space that hardly any neurons become active for multiple object positions. This problem worsens when CT instead of trace learning is used, because any information carried along the temporal dimension is neglected.

Hand-centred visual representations in the primate brain are thought to be used for accurately guiding hand movements towards target objects in the environment. Visually guided reaching, itself, is a highly complex process whereby visual information is transformed into motor actions with a feedback loop for online correction of movement. This paper does not attempt to model such full motor processes. Instead, we have focused on modelling how the hand-centred visual representations, themselves, might arise in a self-organizing fashion using a biologically plausible, unsupervised learning mechanism. The importance of hand-centred visual representations has recently been demonstrated in cognitive robotics by Juett and Kuiper [55]. In their model, self-learned peri-hand representations arose naturally from random exploration and enabled a Baxter Robot to reach to target locations within its peri-personal space. However, Juett and Kuiper used visual information from multiple spatial angles and were not primarily concerned with the biological plausibility of their model architecture, while our model focuses on the development of hand-centred visual neurons within a biologically detailed, hierarchical neural network model of the primate visual cortex. Indeed, in future work we plan to supply the VisNet model with stereoscopic visual input from a simulated 3-dimensional visual training environment, in which the hand is seen with nearby objects. In theory, this could lead to the development of hand-centred neurons that represent 3-dimensional hand-object spatial configurations irrespective of the position of the configuration in 3D peri-personal space.

A key issue investigated in this paper was the performance of the model when trained on a (near) continuum of hand-centred target object locations, as humans are exposed to in every day life. In the simulations reported in section 3.4 we found that the network continued to develop lots of perfect hand-centred output neurons. Moreover, we found that the receptive fields of this population of hand-centred neurons were uniformly distributed throughout the space around the hand, thus providing an even neuronal representation of this hand-centred space. These simulation results lend strong support to our hypothesised role for CT learning in the development of hand-centred visual neurons in the primate brain.

However, we were still left with the fundamental question of why in practice the CT learning mechanism, which builds invariant neuronal responses by simply exploiting spatial overlap between similar input stimuli, encouraged individual output neurons to respond to particular hand-object configurations invariantly across the retinal space, rather than forcing neurons to respond invariantly across the continuum of hand-centred target locations at particular retinal locations. This question was addressed in section 3.5. Based on the close relationship between Oja’s learning rule [51] and the Hebbian learning rule Eq (7) with synaptic weight vector normalisation used in VisNet, it was hypothesised that the output neurons in our model were carrying out an approximate form of Principal Component Analysis (PCA). That is, individual output neurons would learn to represent input patterns with correspondence to the largest sources of variation within the stimulus set used to train the network. In this case, the lateral inhibition between output neurons in our model will likewise drive them to be sensitive to the hand-centred locations of objects rather than the location of hand-object configurations on the retina. The plausibility of this explanation was investigated by performing PCA on the stimulus sets used in a number of different simulations. Consistent with our theoretical explanation for the development of hand-centred neurons when the model is trained on a (near) continuum of target locations around the hand, it was indeed found that the first eigenimages of the stimulus sets used in simulations that developed hand-centred neurons represented the locations of target objects with respect to the hand.

Fig 10 presented two simulations, shown in rows (d) and (f), which failed to develop perfect hand-centred output neurons. In these simulations, the only difference was that the hand-object configurations were presented shifting across a larger horizontal region of retinal space. So what biological features might still be missing in our network model that might improve its ability to develop hand-centred neurons? In future work we intend to upgrade the biological realism of the model and investigate the effects of these modifications on model performance. These planned model improvements concern alterations to the bottom-up visual processing and the incorporation of top-down tactile and proprioceptive signals as follows.

Firstly, let us consider the bottom-up visual processing within the existing model. We have not yet implemented a cortical magnification factor within the hierarchy of cortical layers within the network model, whereby the representation of a visual stimulus close to the fovea is enhanced compared to more peripheral vision. Neither have we attempted in this paper to model active fixation, that is, how a human subject actually shifts their eyes around visual scenes containing their hands with background objects. Although the feasibility of this approach has been demonstrated in an earlier study, in which we showed that hand-centred visual neurons still developed using trace learning when the model was fed with realistic visual input inferred from experimentally recorded human gaze changes based on an eyetracking system [35]. Introducing these improvements to the biological accuracy of the model might perhaps reduce the region of retinal space over which the network learns translation invariant responses to hand-object configurations but also make the development of hand-centred neurons more robust.

Secondly, motion-encoding neurons from the dorsal stream (e.g. MT), that can predict the future position of a target before it is actually reached, could pre-activate those hand-centred cells with the expected target position in their receptive field. Indeed, motion signals seem to be encoded in different reference frames, suggesting that visual decisions for action consider motion in a frame of reference best suited for the current task [56]. Thus, early sub-threshold stimulation from speed-encoding neurons may help to overcome the current limitation of our model regarding the retinal space along which a particular hand-object configuration is shifted.

Thirdly, the current model does not incorporate top-down tactile or proprioceptive signals. For example, neuronal areas preparing and guiding reaching movements receive afferent signals from the somatosensory cortex representing the touch of an object against part of the limb/hand [57], thus indicating the hand-centred location of a target object. Consistent with this, it has been found that some hand-centred or peri-hand neurons display bimodal visual/tactile receptive fields, responding to either the sight or touch of an object in a particular position with respect to the hand. Adding tactile signals to the model may allow us, for example, to account for the bimodal properties of some hand-centred receptive fields as shown previously by Magosso et al. [31, 32]. More recently, Pitti et al. [58] integrated tactile information from an artificial skin with visual information in a recurrent spiking neural network model and even accounted for a successful replication of the rubber-hand illusion. However, these studies do not particularly address the problem of coordinate transforms in multisensory processing nor do they incorporate proprioceptive information in their model.

In addition, reach related areas receive somatosensory signals carrying proprioceptive information about the position of the limb/hand in peri-personal space [59]. Again, consistent with this, hand-centred neurons in posterior parietal cortex can maintain their firing behavior in absence of visual information [16], and can guide movement of the hand using proprioceptive information alone. Tactile and proprioceptive information could play an important role in guiding the development of hand-centred neurons in the brain. Introducing such signals into the model architecture may also lead to more robust development of hand-centred neurons.
